# ﻿Synopsis of Chinese *Alloperla* Banks, 1906 (Plecoptera, Chloroperlidae), with description of a new species

**DOI:** 10.3897/zookeys.1243.139128

**Published:** 2025-07-01

**Authors:** Abdur Rehman, Qing-Bo Huo, Yu-Zhou Du

**Affiliations:** 1 College of Plant Protection & Institute of Applied Entomology, Yangzhou University, Yangzhou 225009, China Yangzhou University Yangzhou China; 2 Joint International Research Laboratory of Agriculture and Agri-Product Safety, the Ministry of Education, Yangzhou University, Yangzhou 225009, China Yangzhou University Yangzhou China

**Keywords:** *Alloperlatriquetra* sp. nov., identification key, new record, redescription

## Abstract

A new species of the genus *Alloperla* Banks, 1906 (Plecoptera, Chloroperlidae), *Alloperlatriquetra* Rehman, Huo & Du, **sp. nov.**, is described from Liaoning Province, China, with diagnostic features and color images of the adult habitus included. The terminalia of the new species are compared with those of related species, and key similarities and distinguishing characteristics are highlighted. In addition, the male of *A.joosti* Zwick, 1972 is described from China for the first time, as previous records included only females. Detailed color photographs of the dorsal and ventral views of the epiproct are provided, addressing a gap in the literature where illustrations of the ventral view were previously lacking a critical feature for accurate species identification. Furthermore, color illustrations and supplementary redescriptions of *A.mediata* (Navás 1925) and *A.teleckojensis* Šámal, 1939 from Liaoning Province, China are presented. An updated checklist and identification key to males of Chinese *Alloperla* species are also provided.

## ﻿Introduction

The genus *Alloperla* Banks, 1906 belongs to the subfamily Chloroperlinae of the family Chloroperlidae. *Alloperla* includes about 52 recognized species distributed across the Nearctic and Palearctic realms (DeWalt et al. 2025). In China, eight species have been documented: *Alloperlamediata* (Navás 1925), *A.erectospina* Wu, 1938, *A.thompsoni* Nelson & Hanson, 1968, *A.joosti* Zwick, 1972, *A.yangi* Li & Wang, 2011, *A.kurentzovi* Zhiltzova & Zapekina-Dulkeit, 1977, *A.kurilensis* Zhiltzova, 1978, and *A.teleckojensis* Šámal, 1939 (Navás 1925; Wu 1938; Nelson and Hanson 1968; Li and Wang 2011; Huo et al. 2022; Zhang et al. 2024). Among these, *A.kurentzovi*, *A.kurilensis* (Zhang et al. 2024), and *A.joosti* (Huo et al. 2022) have been reported only from female specimens, with no males yet collected.

*Alloperla* species in China have primarily been found in Heilongjiang, Liaoning, Inner Mongolia, and Gansu provinces which border on Mongolia and Russia. Although *Alloperla* has mainly been recorded from northeastern and north-central China, its actual distribution may be broader. This possibility is supported by the wider distribution patterns of related green stonefly genera, such as *Sweltsa* Ricker, 1943, *Suwallia* Ricker, 1943, and *Haploperla* Navás, 1934, which are more widely distributed and have also been reported from southwestern China (Rehman et al. 2022a, 2022b, 2022c). Southwestern China, characterized by diverse habitats and climates, provides suitable conditions for many stonefly species, suggesting that the limited records of *Alloperla* may be due to insufficient sampling rather than true absence. To address these gaps, the present study provides new records and a comprehensive synopsis of the genus *Alloperla* in China based on newly collected specimens. Our findings contribute to clarifying the taxonomy, enhancing species identification, and expanding the known distribution of *Alloperla* within the country.

## ﻿Materials and methods

All specimens were collected using aerial nets or by hand and preserved in 75% ethanol. Terminalia were examined and illustrated with KEYENCE VHX-5000 and the final images were prepared using Adobe Photoshop. The type specimens of the new species were deposited in the insect collection of Yangzhou University (**ICYZU**), Jiangsu Province, China. Data for the key and distribution details were gathered from published literature (Navás 1925; Wu 1938; Nelson and Hanson 1968; Li and Wang 2011; Huo et al. 2022; Zhang et al. 2024).

## ﻿Results

### ﻿ Alloperla triquetra

Taxon classificationAnimaliaPlecopteraChloroperlidae

Rehman, Huo & Du sp. nov.

6129EECF-AFED-5929-B618-615339A10C43

https://zoobank.org/A2F9297C-C402-4A23-8137-D63CA4D4044C

Figs 1 , 2 , 3

#### Type material.

***Holotype***, • 1 male China, Xiaodonggou Village, Benxi Autonomous County, Liaoning Province, 9–10-VI-2019, 589 m alt., 41°10.806'N, 124°40.148'E, leg. Huo Qing-Bo, Yang Yu-Ben (ICYZU). ***Paratypes***, • 4 males and 6 females (ICYZU), are the same as the holotype.

#### Diagnosis.

**Figure 1.**
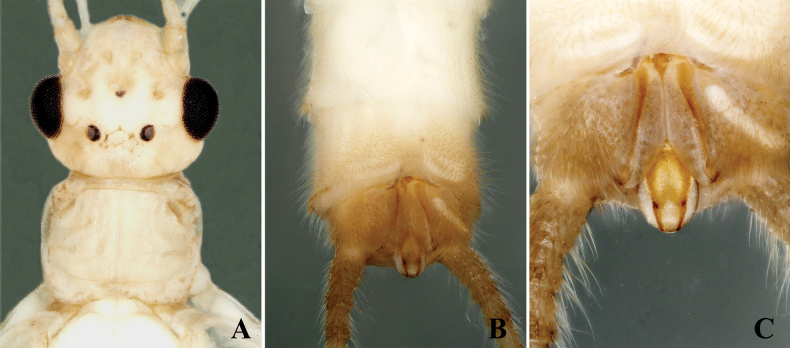
*Alloperlatriquetra* sp. nov. holotype male. **A.** Head and prothorax, dorsal view; **B.** Terminalia, dorsal view; **C.** Epiproct, dorsal view.

**Figure 2.**
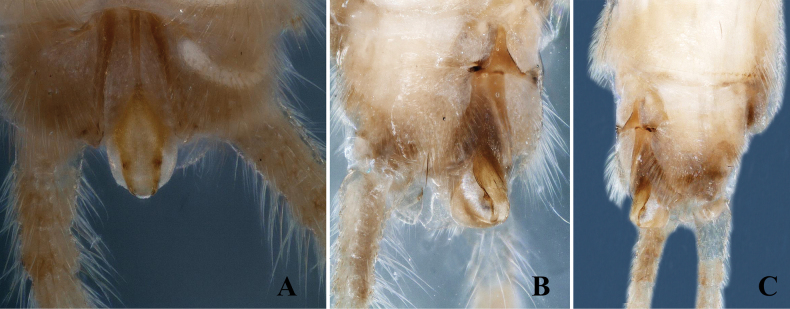
*Alloperlatriquetra* sp. nov. paratype male. **A.** Epiproct, dorsal view; **B.** Epiproct, dorsolateral view; **C.** Terminalia, lateral view.

The new species is characterized by a triangular epiproct. The epiproct is distinctly triangular, especially in its middle portion. Basally, it is narrow and broadens medially before tapering towards a pointed apex, forming a triangular shape. The apex is pointed and bears tiny anterior hairs (Figs 1B, C, 2A).

#### Description.

**Male.** Forewing length: 8.5–9.0 mm; hindwing length: 7.5–8.0 mm. Body greenish in the field, pale yellow in ethanol. Head rounded brown with small central brown markings or spots. Compound eyes blackish, ocelli gray with black margins. Antennae brown, with pale brown basal segments (Fig. 1A). Wings macropterous, hyaline. Abdominal terga 1–6 with a small brown median stripe. Tergum 9 dark brown posteriorly; tergum 10 dark brown, divided medially by the epiproct and reduced cowl into two hemitergal lobes bearing dense matting of long setae (Fig. 1B). Epiproct small, triangular, with a dorsal surface covered by dense, small hairs. Basally narrow, widening medially to a triangular shape with a pointed apex (Fig. 1C). The ventral surface is flattened, broad at the base, tapering towards apex, and bending towards tergum 10 to form a hook-like structure (Fig. 2B, C). Laterally flattened, bearing small hairs. Cerci dark brown, generally brown with small basal segments covered with long setae.

**Figure 3.**
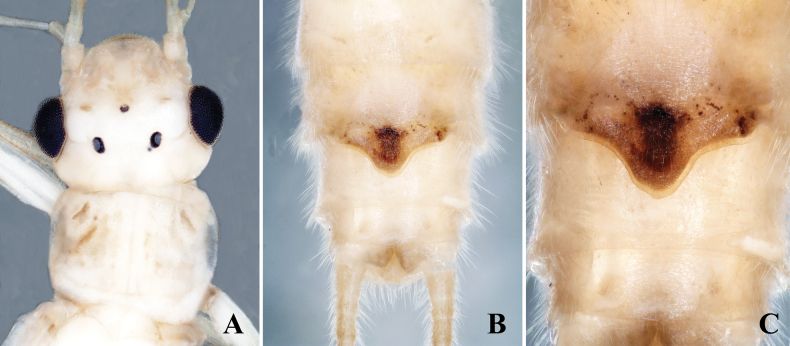
*Alloperlatriquetra* sp. nov. female. **A.** Head and prothorax, dorsal view; **B.** Terminalia, ventral view; **C.** Subgenital plate.

**Female.** Forewing length: 9.0–9.5 mm; hindwing length: 8.0–8.5 mm. Body appears greenish in life and brown in ethanol. Head rounded, brown, with small brown markings or spots on frons. Compound eyes blackish, ocelli gray with black margins. Pronotum brown with slight rugosities (Fig. 3A). Subgenital plate parallel-sided, rectangular at base, then abruptly narrows in middle, forming long subtriangle extending to middle of sternum 9. Posterior margins of plate rounded, and subgenital plate darkly sclerotized with black triangular spot in center (Fig. 3B. C).

**Egg and nymph.** Unknown.

#### Distribution.

Known only from the type locality.

#### Etymology.

The species is named for its epiproct, which is distinctly triangular in shape. The Latin word “*triquetra*” means triangular.

#### Remarks.

The new species is most similar to *Alloperlayangi* Li & Wang, 2011, from Quanshanlinchang, Kuandian County, Liaoning, China, but it is easily distinguished by the structure of the epiproct. The epiproct of the new species is triangular with a pointed apex (Figs 1B, 2A). In contrast, the epiproct of *A.yangi* is tongue-shaped, rounded at the apex, and bears small apical setae (Li and Wang 2011: figs 1–9). The new species is also similar to *A.teleckojensis* Šámal, 1939, which is widely distributed in Russia and Mongolia. However, the epiproct of the new species is more prominent and broader than *A.teleckojensis* (Zhang et al. 2024: figs 92–95).

##### ﻿Checklist of the named *Alloperla* known from China

***Alloperla* Banks, 1906**

*Alloperla* Banks, 1906:175.

###### ﻿*Alloperlamediata* (Navás, 1925)

*Chloroperlamediata* Navás, 1925: 210; Claassen 1940: 191; Illies 1966: 441.

Alloperla (Sweltsa) mediata (Navás, 1925): Nelson and Hanson 1968: 425.

*Alloperlaalexanderi* Nelson & Hanson, 1968: Zhiltzova and Zwick 1971.

*Alloperlamediata* (Navás, 1925) in Zwick 1973: 285; Ham 2008: 185; Teslenko 2009: 699; Judson and Nelson 2012: 25; Huo et al. 2022: 9.

**Material examined.** • 2 females, China: Inner Mongolia Autonomous Region, HulunBuir city, Oroqen Zizhiqi, Dayangshu town, 405 m, 49.7086°N, 124.5899°E, 2023.VI.3, leg. Zhu Ya-Fei & Yang Xiao (ICYZU) • 1 male, 5 females, Heilongjiang Province, Da Hinggan Ling Prefecture, Songlin District, Jinsong town, 480 m, 51.0724°N, 124.1948°E, 2023.VI.4, leg. Zhu Ya-Fei & Yang Xiao; • 3 males, 20 females • Heilongjiang Province, Da Hinggan Ling Prefecture, Xinlin District, Dawusu town, 471 m, 51.7682°N, 124.5122°E, 2023.VI.6, leg. Zhu Ya-Fei & Yang Xiao (ICYZU); • 4 males, 2 females China: Heilongjiang Province, Da Hinggan Ling Prefecture, Tahe county, 364 m, 52.3050°N, 124.6968°E, 2023.VI.6, leg. Zhu Ya-Fei & Yang Xiao (ICYZU).

**Figure 4.**
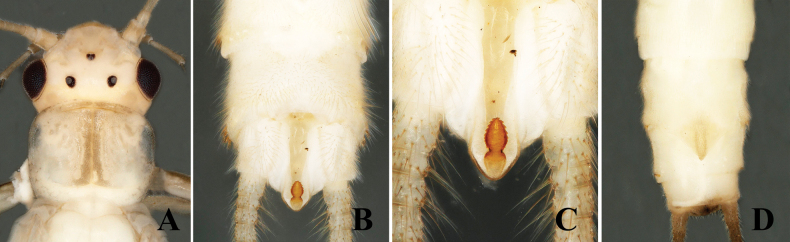
*Alloperlamediata* (Navás, 1925) Male. **A.** Head and prothorax, dorsal view; **B.** Terminalia, dorsal view; **C.** Epiproct, dorsal view; **D.** Female terminalia, ventral view.

**Supplementary description.** General body color pale yellow in alcohol. Head pale brown, triocellate. Pronotum with prominent, dark-brown medial stripe (Fig. 4A). Epiproct short, pointed, divided into two portions, nearly equal in width, separated by noticeable constriction. Anterior portion of epiproct oval, bearing several spines along lateral margins, easily visible from dorsal view (Fig. 4B, C). Female subgenital plate long, narrowly triangular with rectangular, parallel-sided base, extending nearly full length of sternum 9 (Fig. 4D).

**Distribution.** China (Heilongjiang, Inner Mongolia, and Liaoning), Japan, Mongolia, Russia, and South Korea.

**Remarks.** This species was first described from China by Nelson and Hanson (1968) as *Alloperlaalexanderi* Nelson & Hanson, 1968, but it was later synonymized with *A.mediata* by Zhiltzova and Zwick (1971). The type locality is the Russian Far East, which includes Khabarovsk and Vladivostok. It has been recorded in China from Heilongjiang, Inner Mongolia, and the Greater Khingan Mountains.

###### ﻿*Alloperlaerectospina* Wu, 1938

*Alloperlaerectospina* Wu, 1938: 152; Claassen 1940: 186; Illies 1966: 431; Wu 1973: 100; Du and Sivec 2005: 42; Yang and Li 2018: 23.

**Distribution.** China (Gansu Province).

**Remarks.**Wu (1938) described this species based on a single male specimen, with the female still unknown. The type locality is Mahoshan Mountain in Gansu, China. Currently, this species is known only from Gansu Province, China.

###### ﻿*Alloperlathompsoni* Nelson & Hanson, 1968

Alloperla (Sweltsa) thompsoni Nelson & Hanson, 1968: 425.

*Alloperlathompsoni*: Zwick 1973: 287; Stark and Sivec 2009: 156; Yang and Li 2018: 22.

**Distribution.** China (Heilongjiang Province).

**Remarks.**Nelson and Hanson (1968) described this species based solely on a male specimen, with the female still unknown. The type locality is Yalu Station in the Greater Khingan Mountains, Manchuria, China. Currently, this species is only known from Heilongjiang Province, China.

###### ﻿*Alloperlakurilensis* Zhiltzova, 1978

*Alloperlakurilensis* Zhiltzova, 1978: 544; Zhiltzova 1995: 15; Teslenko 2009: 699.

**Distribution.** China (Liaoning Province) and Russia.

**Remarks.**Zhang et al. (2024) newly reported this species from China (from Quanshan Forest Farm in Kuandian Manchu Autonomous County, Dandong City, Liaoning Province), based only on a female specimen; the male is still undescribed in China. The type locality is Kunashir, Russia (DeWalt et al. 2025).

###### ﻿*Alloperlakurentzovi* Zhiltzova & Zapekina-Dulkeit, 1977

*Alloperlakurentzovi* Zhiltzova & Zapekina-Dulkeit, 1977: 50; Zhiltzova 1995: 15; Teslenko 2009: 699.

**Distribution.** China (Liaoning Province) and Russian Far East.

**Remarks.**Zhang et al. (2024) newly reported this species from China (from Quanshan Forest Farm in Kuandian Manchu Autonomous County, Dandong City, Liaoning Province), based solely on a female specimen; the male is still undescribed from China. The type locality is the Russian Far East, Primorye (DeWalt et al. 2025).

###### ﻿*Alloperlateleckojensis* Šámal, 1939

*Alloperlateleckojensis* Šámal, 1939: 423.

*Chloroperlateleckojensis*: Illies 1966: 443.

*Alloperlademinuta* Zapekina-Dulkeit, 1970: 159: Zwick 1973: 284; Zhiltzova 1995: 15; Teslenko 2009: 699; Teslenko and Zhiltsova 2009: 89; Judson and Nelson 2012: 24.

**Material examined.** • 1 male, China: Heilongjiang Province, Da Hinggan Ling Prefecture, Tahe county, 364 m, 52.3050°N, 124.6968°E, 2023.VI.6, leg. Zhu Ya-Fei & Yang Xiao (ICYZU).

**Figure 5.**
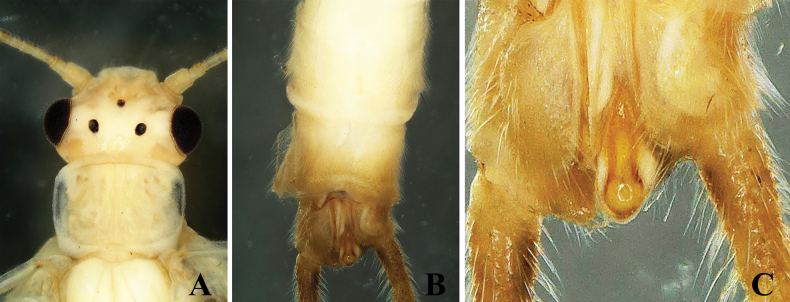
*Alloperlateleckojensis* Šámal, 1939 male. **A.** Head and prothorax, dorsal view; **B.** Terminalia, dorsal view; **C.** Epiproct dorsal view.

**Supplementary description.** Male body pigmentation matches previous descriptions in the literature. Tergum X divided and features two parallel sclerotized bands along inner margin. Base of epiproct rounded and shiny, with slight constriction near base. In dorsal view, it narrows to form pointed apex, while laterally completely flattened (Fig. 5A–C).

**Distribution.** China (Heilongjiang Province), Russia, and Mongolia.

**Remarks.**Zhang et al. (2024) newly reported this species from China based on male and female specimens from Tahe County in Heilongjiang Province. The type locality is Teletskoye Lake, near the confluence of the Koksa River in the Altai Republic, Russia.

###### ﻿*Alloperlajoosti* Zwick, 1972

*Alloperlajoosti* Zwick, 1972: 35; Ham 2008: 186; Teslenko 2009; Judson and Nelson 2012: 24; Huo et al. 2022: 9.

**Material examined.** • 5 males, 30 females, China: Inner Mongolia Autonomous Region, HulunBuir city, Oroqen Zizhiqi, Dayangshu town, 405 m, 49.7086°N, 124.5899°E, 2023.VI.3, leg. Zhu Ya-Fei & Yang Xiao • 4 females (ICYZU), China: Heilongjiang Province, Da Hinggan Ling Prefecture, Tahe County, 364 m, 52.3071°N,124.7051°E, 2023.VII.30, leg. Zhu Ya-Fei & Yang Xiao.

**Figure 6.**
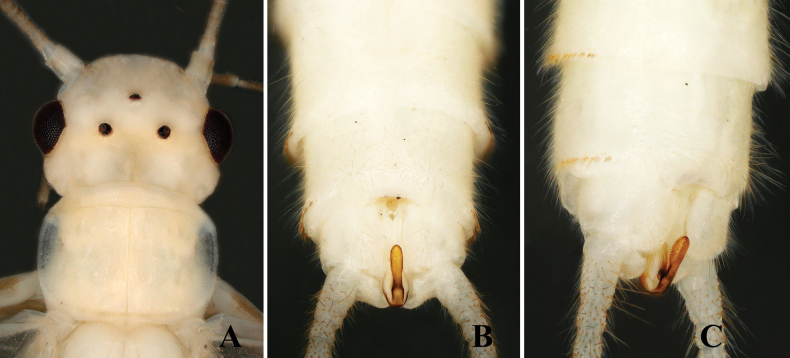
*Alloperlajoosti* Zwick, 1972 Male. **A.** Head and prothorax, dorsal view; **B.** Terminalia, dorsal view; **C.** Terminalia, dorsolateral view.

**Figure 7.**
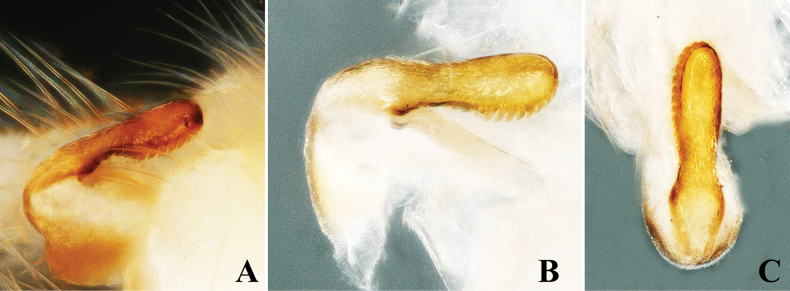
*Alloperlajoosti* Zwick, 1972 Male. **A.** Epiproct, lateral view; **B.** Epiproct lateral view; **C.** Epiproct, dorsal view.

**Supplementary description. Male.** Forewing length: 8.0–8.5 mm; hindwing length: 7.0–7.5 mm. Body greenish in field, pale yellow in ethanol. Head rounded, pale brown without markings or spots. Compound eyes blackish, ocelli gray with black margins. Antennae brown, with pale brown basal segments. Wings macropterous, hyaline. Meso- and metanota with faint pigmentation, no markings or stripes (Fig. 6A). Abdominal terga with prominent dorsal stripe originating on segment 1, continuing through segment 7. Epiproct recurved anteriorly, slightly swollen at base, medially constricted, with black medial stripes anteriorly; uniformly broad toward apex, apex rounded, length approximately four times width (Fig. 6B). Ventrally, epiproct has a row of long spines along margin, medially constricted (Fig. 7A–C). Cerci dark brown, generally brown, small basal segments covered with long setae.

**Figure 8.**
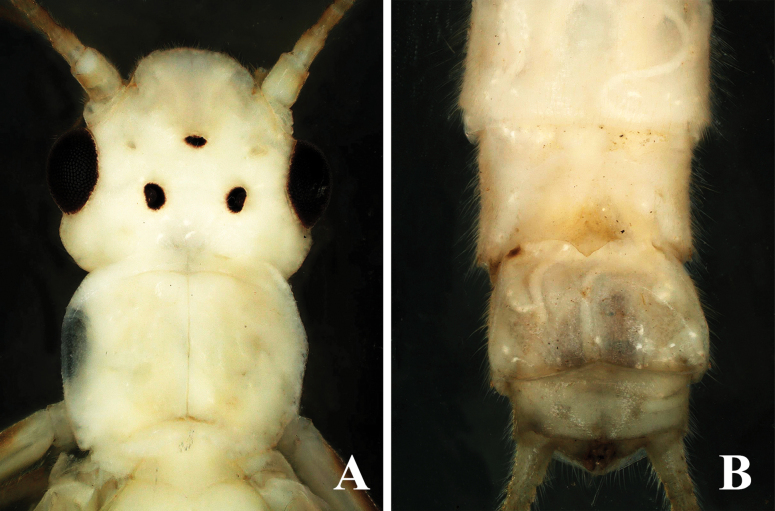
*Alloperlajoosti* Zwick, 1972 female. **A.** Head and prothorax, dorsal view; **B.** Terminalia ventral view.

**Female.** The female subgenital plate is small and V-shaped, extending only to the middle part of sternum 9. The plate gradually slopes to an abrupt point, forming a triangle with a broad base (Fig. 8A, B). The cerci are pale brown.

**Distribution.** China (Inner Mongolia Autonomous Region and Heilongjiang Province), Mongolia, Russia, and South Korea.

**Remarks.**Huo et al. (2022) newly reported this species in China from Xiaodong Gou Village, Benxi County, Liaoning Province, based on female specimens, without describing the male. Here, we provide the first description of the male specimens of this species from China. The species was described initially from magnolia in Russia (Judson and Nelson 2012; Teslenko and Zhiltzova 2009), but neither description included the ventral view of the epiproct (Judson and Nelson 2012: figs 96–98; Teslenko and Zhiltzova 2009: fig. 557), which is essential for identifying this species because it possesses a row of spines in the ventral view. We offer a revised description with clear color images of both dorsal and ventral views to avoid future misidentifications. The ventral view of the epiproct bears a distinct row of spines (Fig. 7A, B). Additionally, we observed variation in the subgenital plate among some female individuals; some have a short, sloped structure while others have a little longer extension. Earlier literature indicates that the figures provided for the female subgenital plate in Judson and Nelson (2012) and Teslenko and Zhiltzova (2009) differ from each other, suggesting that variation in the female subgenital plate is common in this species. The type locality of this species is Arkhangai Aimag, Somon Chencher at Urd-Tamir Gol, Mongolia (Judson and Nelson 2012).

###### ﻿*Alloperlayangi* Li & Wang, 2011

*Alloperlayangi*Li and Wang 2011: 29; Yang and Li 2018: 22.

**Distribution.** China (Liaoning Province).

**Remarks.**Li and Wang (2011) described only the male of this species, and the female remains unknown. The type locality is Quanshanlinchang, Kuandian County, Liaoning. This species is currently known only from Liaoning Province, China.

### ﻿Key to adult males of *Alloperla* species from China

**Table:** 

1	Epiproct small, flattened, or constricted in dorsal view	**2**
–	Epiproct long, rounded, or flattened in dorsal view	**6**
2	Epiporct flattened in dorsal view, with dorsum appressed setae	**3**
–	Epiproct constricted medially in dorsal view	**4**
3	Epiproct tongue-shaped, apically rounded with shallow marginal serrations (Li and Wang 2011: figs 1–11)	***A.yangi***
–	Epiproct triangular, apically pointed without marginal serrations; lateral margins covered with scattered hairs (Figs 1, 2)	***A.triquetra* sp. nov.**
4	Epiporct divided or constricted medially; anterior portion of epiproct oval, bearing several spines along lateral margins (Fig. 4)	***A.mediata***
–	Epiproct round or triangular without any constriction medially; anterior portion without spines	**5**
5	Epiproct completely rounded without marginal appressed setae (Nelson and Hanson 1968: figs 1–3)	***A.thompsoni***
–	Epiproct triangular, covered with setae on margins and upturned in lateral view (Wu 1938: figs 182, 183)	***A.erectospina***
6	Epiporct flattened, long and narrow apically, apex pointed like pen, laterally flattened (Fig. 5)	***A.teleckojensis***
–	Epiporct long, rounded, and broader apically, ventrally with a arow of spines (Figs 6, 7)	***A.joosti***

## Supplementary Material

XML Treatment for Alloperla triquetra

## ﻿Additional information

### Author ORCIDs

Abdur Rehman https://orcid.org/0000-0001-7379-6482

Qing-Bo Huo https://orcid.org/0000-0001-9197-8503

Yu-Zhou Du https://orcid.org/0000-0002-4452-7125

### Data availability

All of the data that support the findings of this study are available in the main text.
